# The Short-Term Effect of Ambient Temperature on Mortality in Wuhan, China: A Time-Series Study Using a Distributed Lag Non-Linear Model

**DOI:** 10.3390/ijerph13070722

**Published:** 2016-07-18

**Authors:** Yunquan Zhang, Cunlu Li, Renjie Feng, Yaohui Zhu, Kai Wu, Xiaodong Tan, Lu Ma

**Affiliations:** 1 Department of Epidemiology and Biostatistics, School of Public Health, Wuhan University, Wuhan 430071, China; Yun-quanZhang@whu.edu.cn (Y.Z.); lanxilzu@126.com (C.L.); 2014203050012@whu.edu.cn (R.F.); 13349888239@163.com (Y.Z.); 2 Jiang’an District Center for Disease Control and Prevention, Wuhan 430014, China; uueng@hotmail.com; 3 Department of Occupational and Environmental Health, School of Public Health, Wuhan University, Wuhan 430071, China; xiaodongtan@yahoo.com

**Keywords:** climate change, temperature, mortality, distributed lag non-linear model

## Abstract

Less evidence concerning the association between ambient temperature and mortality is available in developing countries/regions, especially inland areas of China, and few previous studies have compared the predictive ability of different temperature indictors (minimum, mean, and maximum temperature) on mortality. We assessed the effects of temperature on daily mortality from 2003 to 2010 in Jiang’an District of Wuhan, the largest city in central China. Quasi-Poisson generalized linear models combined with both non-threshold and double-threshold distributed lag non-linear models (DLNM) were used to examine the associations between different temperature indictors and cause-specific mortality. We found a U-shaped relationship between temperature and mortality in Wuhan. Double-threshold DLNM with mean temperature performed best in predicting temperature-mortality relationship. Cold effect was delayed, whereas hot effect was acute, both of which lasted for several days. For cold effects over lag 0–21 days, a 1 °C decrease in mean temperature below the cold thresholds was associated with a 2.39% (95% CI: 1.71, 3.08) increase in non-accidental mortality, 3.65% (95% CI: 2.62, 4.69) increase in cardiovascular mortality, 3.87% (95% CI: 1.57, 6.22) increase in respiratory mortality, 3.13% (95% CI: 1.88, 4.38) increase in stroke mortality, and 21.57% (95% CI: 12.59, 31.26) increase in ischemic heart disease (IHD) mortality. For hot effects over lag 0–7 days, a 1 °C increase in mean temperature above the hot thresholds was associated with a 25.18% (95% CI: 18.74, 31.96) increase in non-accidental mortality, 34.10% (95% CI: 25.63, 43.16) increase in cardiovascular mortality, 24.27% (95% CI: 7.55, 43.59) increase in respiratory mortality, 59.1% (95% CI: 41.81, 78.5) increase in stroke mortality, and 17.00% (95% CI: 7.91, 26.87) increase in IHD mortality. This study suggested that both low and high temperature were associated with increased mortality in Wuhan, and that mean temperature had better predictive ability than minimum and maximum temperature in the association between temperature and mortality.

## 1. Introduction

Global climate change has become a growing public health concern in recent years. As one of the biggest global health threats in the 21st century [1,2], climate change has led to an increased frequency and intensity of extreme temperatures [3], especially increased heat wave events. Moreover, health impacts due to climate change will continue to occur, at least for the foreseeable future [4]. Therefore, it is of great significance and urgency for decision makers to develop adaptive strategies to climate change based on local cold- and heat-related epidemiological studies.

A non-linear U, J, or V-shaped relationship between temperature and mortality has been identified in many previous studies, which revealed the adverse effects of low and/or high temperatures [5,6,7]. However, the associations between temperature and mortality varied by geographic locations, study populations, and climatic characteristics [8,9,10]. Thus, it is quite necessary for different regions to conduct locally-based environmental epidemiology studies when assessing temperature-related mortality impact for local public health decision-making.

People in developing countries are anticipated to be susceptible to the impact of extreme temperatures because of more limited adaptive capacity and more vulnerable than people in developed countries [2,11]. Nevertheless, current knowledge of health effects of temperature on mortality is mainly from developed countries, and only a limited number of studies have been conducted in developing countries [3]. In China, mortality impact assessment of extreme temperatures has got increasing attention in recent years and relevant studies have been conducted in several large cities in southeastern coastal areas, like Beijing [12,13], Shanghai [14], Guangzhou [15,16], Suzhou [3], and Tianjin [17]. However, to date, less studies have been reported in Chinese inland areas, including several major cities in central China. Moreover, previous studies usually used mean temperature as the temperature metric, few of them have compared the effects of different temperature indicators on mortality.

As the largest city in central China, Wuhan has a climate characterized by cold winters and extremely hot summers. In order to help establishing local warning and preventive control systems to reduce both cold- and heat-related adverse health effects, the present study assessed the locally-based association between ambient temperature and cause-specific mortality and evaluated the predictive ability of different temperature indicators (minimum, mean, and maximum) on mortality in Wuhan, China.

## 2. Materials and Methods

### 2.1. Study Area and Population

Wuhan, the largest city in central China, is located in the middle of the Yangzi River, at 29°58′–31°22′ north latitude and 113°41′–115°05′ east longitude. Wuhan has a typical subtropical monsoon climate with a distinct pattern of four seasons. Jiang’an District is one of the seven major central urban districts in Wuhan and was the political, economic, cultural, financial, and information center of Wuhan City. The population of permanent residents in Jiang’an District was about 0.68 million in 2010 and urban area was 64.24 km^2^. In 2010, females accounted for 50.2%, and the elderly (age ≥ 65 years old) accounted for 12.7% of the total population.

### 2.2. Data Collection

Daily mortality data from 1 January 2003 to 31 December 2010 were obtained from Centre for Disease Control and Prevention of Jiang’an District in Wuhan, China. The causes of death were coded according to the 10th Revision of the International Classification of Disease (ICD10). The mortality data were stratified into five cause-specific categories: non-accidental mortality (A00–R99), cardiovascular mortality (I00–I99), respiratory mortality (J00–J99), stroke mortality (I60–I69), and ischemic heart disease (IHD) mortality (I20–I25).

Daily meteorological data on maximum, mean, and minimum temperature and relative humidity during the study period were obtained from the China Meteorological Data Sharing Service System [18]. Daily air pollution data on particulate matter <10 μm in aerodynamic diameter (PM_10_), sulfur dioxide (SO_2_), and nitrogen dioxide (NO_2_) were collected from the Wuhan Environmental Monitoring Center.

### 2.3. Data Analysis

We applied quasi-Poisson generalized linear models (GLM) because daily deaths typically followed an over-dispersed Poisson distribution [19]. Several covariates were incorporated in the GLM: (1) a natural cubic smooth function of calendar time with 4 degrees of freedom (df) per year to exclude unmeasured long-term and seasonal trends in daily mortality; (2) natural cubic smooth functions of air pollutants (PM_10_, SO_2_, and NO_2_) and relative humidity in accordance with previous studies [17]; and (3) indicator variables for “day of the week (DOW)” and public holiday [20].

Specifically, the degrees of freedom for time trend were selected by minimizing the sum of the absolute value of the partial autocorrelation function (PACF) of the GLM model’s residuals up to 30 lags [21]. This minimization intends to minimize the correlation between residuals from proximate observations in the data series, to match the standard assumption of uncorrelated residuals [5].

In order to flexibly account for the potential lagged and non-linear effects of temperature on mortality, we incorporated temperature as a “cross-basis” function in the GLM, which was constructed using a distributed lag non-linear model (DLNM) [22]. According to the Akaike information criterion (AIC) for quasi-Poisson models [22], we applied a natural cubic spline with 6 df to model the non-linear association between temperature and mortality, and a natural cubic spline with 4 df to model the lagged effect of temperature. The spline knots of temperature and lags were respectively placed at equal spaces and equal intervals in the log scale to allow enough flexibility in the two ends of temperature distribution and lag effects at shorter delays [17,22]. To completely capture the overall temperature effects and adjust for any potential harvesting (i.e., heat-related mortality excess were followed by deficits), we used lags up to 21 days according to a previous study [9]. A temperature of 19.2 °C (which was approximately the median value during the study period) was used as the reference value to calculate the relative risks.

Our initial analysis found that the temperature–mortality relationships were U-shaped, with potential cold and hot thresholds. Thus, we also applied a double-threshold DLNM, assuming the effect of cold temperature was linear below the cold threshold, whereas the effect of high temperature was linear above the hot threshold, and we modeled the lag effects using a natural cubic spline with 4 df. Temperature thresholds were determined by testing multiple thresholds according to two previous studies by examining all the potential combinations of cold thresholds and hot thresholds to identify the combination that minimized the residual deviance [15,17]. For instance, for mean temperature and non-accidental mortality, our initial analysis indicated that the potential cold threshold was within 10 to 20 °C and the potential hot threshold was within 25 to 35 °C. Hence we identified the combination that minimized the residual deviance from combinations of cold thresholds from 10 to 20 °C (by 0.1 °C) and hot thresholds from 25 to 35 °C (by 0.1 °C) using double-threshold DLNM. Cold effect and hot effect were then estimated as the percent increase in mortality for a 1 °C decrease in temperature below the cold threshold and a 1 °C increase above the hot threshold.

To check the main findings of this study, sensitivity analyses were performed by changing df (3–8 per year) in the smoothness of time to control time trend. We also changed df (4–7) for humidity, PM_10_, SO_2_, and NO_2_, and changed the maximum lags from 22 to 30 days in the temperature-DLNMs. Additionally, we conducted age-stratified analyses and several sensitivity analyses using different threshold values for total mortality.

The statistical tests were two-sided, and effects of *p* < 0.05 were considered statistically significant. All the analyses were performed with the R software (version 3.1.3; R Foundation for Statistical Computing, Vienna, Austria) using the package dlnm, version 2.1.3, publicly available on the R comprehensive archive network (CRAN).

## 3. Results

During the study period (1 January 2003–31 December 2010), there were 32,721 non-accidental deaths in total; 15,303 persons (46.8%) died from cardiovascular disease, including 9204 (28.1%) of stroke and 3900 (11.9%) of IHD, and 3081 persons (9.4%) died from respiratory disease (Table 1). An average of 11.2, 5.2, 3.1, 1.3, and 1.1 cases per day occurred for non-accidental, cardiovascular, stroke, IHD, and respiratory mortality, respectively. The average daily maximum, mean, and minimum temperature were 22.2 °C (range: −1.9 °C, 39.6 °C), 17.9 °C (range: −2.7 °C, 35.8 °C), and 14.6 °C (range: −5.2 °C, 32.3 °C), respectively. The average daily relative humidity was 71.3% and ranged from 21.0% to 97.0%. The mean concentrations of daily PM_10_, SO_2_, and NO_2_ were 115.0 μg/m^3^, 50.2 μg/m^3^ and 57.6 μg/m^3^, respectively, which were well above the international health-based standards.

Table 2 shows the Spearman correlation coefficients of weather conditions and ambient pollutants. Three temperature measures were strongly correlated with each other, negatively correlated with ambient pollutants, and moderately correlated with relative humidity. PM_10_, SO_2_, and NO_2_ were strongly correlated with each other.

Table 3 calculates the QAIC values of non-threshold and double-threshold DLNM models predicted by different temperature measures. Double-threshold DLNM models performed better than non-threshold DLNM models with smaller QAIC values, and mean temperature had the best predictive ability for mortality. Therefore, the results of associations between mean temperature and mortality were only reported in this paper. Cold and hot thresholds used by the double-threshold DLNM models were presented in Table 4.

**Figure 1:**
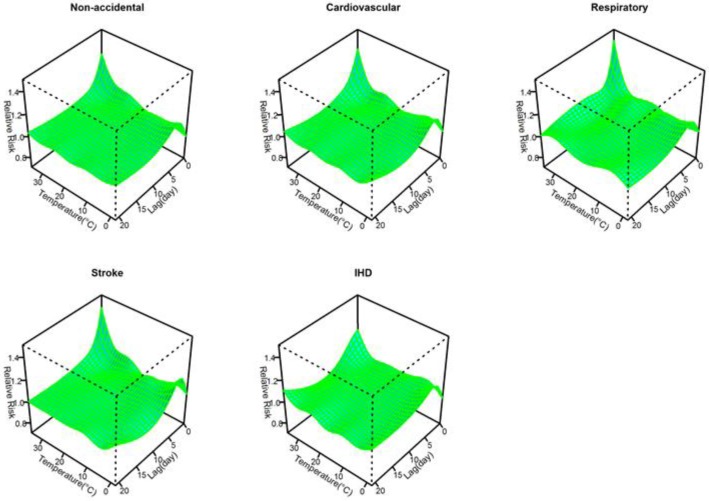
Relative risks of cause-specific mortality by mean temperature (°C) and lag in Jiang’an District of Wuhan, China during 2003 to 2010. The risks used 6 df for temperature and 4 df for lag up to 21 days and the reference temperature was the median temperature (19.2 °C) during the study period.

Figure 1 shows the three-dimensional plots of the relationships between mean temperature and cause-specific mortality along lags of 21 days. The estimated effects of temperature were non-linear for all cause-specific mortality categories, with higher relative risks at hot and cold temperatures, while extremely hot temperatures were associated with much higher relative risks than extremely cold temperatures within a lag of 3 days.

**Figure 2:**
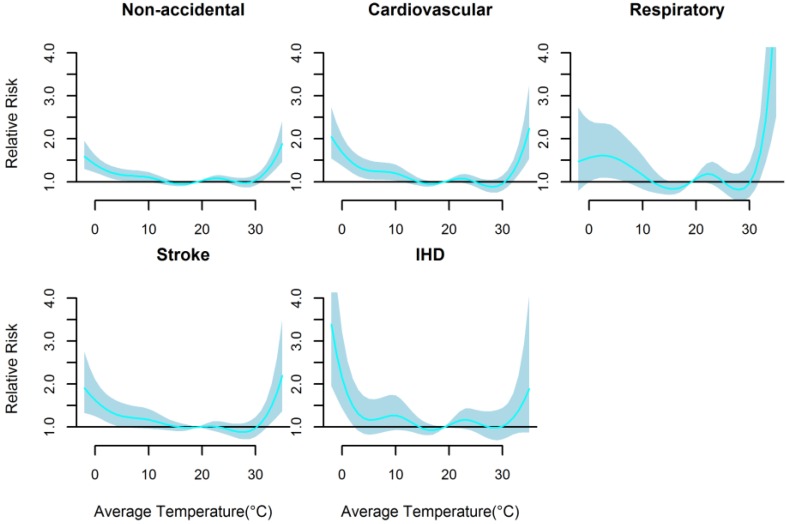
The non-linear effects of mean temperature on cause-specific mortality at lag 0–21, using a DLNM model with 6 df natural cubic spline for temperature and 4 df for lag.

Figure 2 presents the non-linear effects of mean temperature on cause-specific mortality over 21 days. There were U-shaped relationships between mean temperature and all mortality types. The relative risks of all mortality types increased rapidly only when mean temperature were above 30.0 °C (approximately *P*_90_ quantile of the temperature distribution), while increased slowly below 20.2 °C (approximately *P*_54_ quantile of the temperature distribution) except IHD mortality with rapid increase below 3.5 °C. The cold and hot thresholds were 18.1 °C and 31.7 °C for non-accidental mortality, 15.6 °C and 31.4 °C for cardiovascular mortality, 14.6 °C and 31.2 °C for respiratory mortality, and 20.2 °C and 32.2 °C for stroke mortality, and 3.5 °C and 30.0 °C for IHD mortality (Table 4).

**Figure 3:**
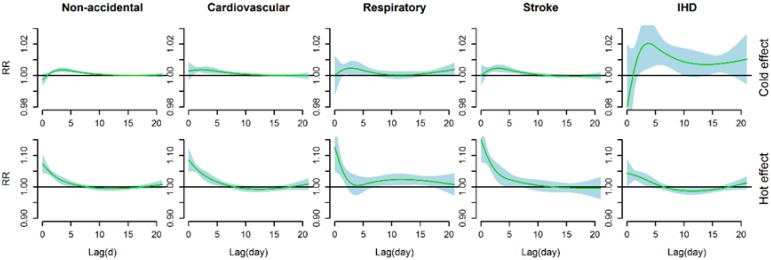
The estimated effects of a 1 °C decrease in mean temperature below the cold threshold (**above**) and of a 1 °C increase in mean temperature above the hot threshold (**below**) on cause-specific mortality over 21 days of lag, using a double-threshold DLNM with 4 df natural cubic spline for lag. The cold and hot thresholds were 18.1 °C and 31.7 °C for non-accidental mortality, 15.6 °C and 31.4 °C for cardiovascular mortality, 14.6 °C and 31.2 °C for respiratory mortality, 20.2 °C and 32.2 °C for stroke mortality, and 3.5 °C and 30.0 °C for IHD mortality.

Figure 3 shows the estimated effects of cold effects (a 1 °C decrease in mean temperature below the cold threshold) and heat effects (a 1 °C increase in mean temperature above the hot threshold) on cause-specific mortality over 21 days of lag. Significant cold effects appeared after a lag of 1–2 days and lasted about 10 days (about 14 days for IHD mortality), whereas significant hot effects occurred immediately and lasted less than a week (about 10 days for respiratory mortality).

We calculated the cumulative effects of mean temperature on cause-specific mortality at different amounts of lag (Table 5). For cold effects over a lag of 0–21 days, a 1 °C decrease in mean temperature below the cold thresholds was associated with a 2.39% (95% CI: 1.71, 3.08) increase in non-accidental mortality, 3.65% (95% CI: 2.62, 4.69) increase in cardiovascular mortality, 3.87% (95% CI: 1.57, 6.22) increase in respiratory mortality, 3.13% (95% CI: 1.88, 4.38) increase in stroke mortality, and 21.57% (95% CI: 12.59, 31.26) increase in IHD mortality. For hot effects over lag 0–7 days, a 1 °C increase in mean temperature above the hot thresholds was associated with a 25.18% (95% CI: 18.74, 31.96) increase in non-accidental mortality, 34.10% (95% CI: 25.63, 43.16) increase in cardiovascular mortality, 24.27% (95% CI: 7.55, 43.59) increase in respiratory mortality, 59.1% (95% CI: 41.81, 78.5) increase in stroke mortality, and 17.00% (95% CI: 7.91, 26.87) increase in IHD mortality. Hot effects over lag of different days were much greater than cold effects consistently except for IHD mortality at lags of longer than 14 days.

We changed df (3–8 per year) for time to control for the season, which gave similar results. We changed df (4–7) for humidity, PM_10_, SO_2_, and NO_2_, and varied the maximum lag from 22 to 30 days, the estimated effects of temperature did not substantially change. In addition, we conducted sensitivity analyses for total mortality using different threshold values, which also gave similar results (Figure S1). Also, age-stratified analyses confirmed our results of relationships between cause-specific mortality and mean temperature (Figures S2 and S3 and Table S1). All in all, the models used in this study seemed to have adequately captured the main effects of temperature on mortality.

## 4. Discussion

In the present study, we examined the effects of ambient temperature on cause-specific mortality in Wuhan, China during the years 2003 to 2010. Non-linear and U-shaped relationshps were consistently found between temperature and all cause-specific mortality categories, which revealed that both cold and high temperarure increased the risk of mortality. Further, our data suggested that the effects of hot temperatures on mortality were much larger than that of cold temperatures. These findings may have important implications for public health policies in Wuhan, China.

The study area of the present study, Wuhan, used to be known as one of the Three Furnaces of China. During the study period 2003 to 2010, Wuhan experienced hot summers (June to August) with an average daily temperature of 28.5 °C, and about 28 high temperature days (daily maximum temperature ≥35 °C) occurred per summer. The present study provided an opportunity to examine the temperature-mortality relationship in the extremely hot areas. Jiang’an District located in the central urban area in Wuhan, with a high population density and relatively concentrated and similar living conditions. Considering these factors, the study focus on mortality impact of temperature with a long period in Jiang’an District, Wuhan City may have less assessment bias compared with studies conducted in large cities with great variation in environmental factors, and thus the results of our study may be more proper and applicable to the local practice.

U-shaped relationships were observed between temperature and cause-specific mortality categories in Wuhan, which indicated that both low and high temperature were associated with increased mortality risk. The results were consistent with some previous studies conducted in China [15,17,23,24] and a systematic evaluation of temperature-mortality relationship in 12 countries/regions [9]. However, according to previous studies, the impacts of temperature on different mortality categories may be less consistent. For instance, both cold and heat effects were obvious for cardiovascular disease mortality, but respiratory mortality was found only associated with high temperatures in two previous studies in Australia [25] and Suzhou, China [3]. In Chiang Mai, Thailand, only cold effects on cardiovascular mortality and heat effect on respiratory mortality were found to be significant [26]. Moreover, the effects of temperature on mortality may vary by locations (such as latitudes, climatic conditions), study periods, study designs, and statistical approaches [8,12,23,27]. In addition, gender, age, educational attainment, and other sociodemographic factors of the study popolation can also modify the relationship between temperature and mortality [27,28,29].

Several previous studies have compared the effects of various temperature indicators on different mortality categories [17,30,31]. It was revealed that maximum, minimum, and mean temperatures had similar predictive ablity due to their strong correlations [17,31]. However, mean temperature was most usually used to assess the association between temperature and mortality because mean temperature can represent the exposure throughout the whole day and night and provide more easily interpreted results within a policy context [3,32]. Our study also suggested that mean temperature performed best for predicting the effects of temperature on cause-specific mortality according to AIC values. Nevertheless, Yu et al. [30] found different age groups and death categories were sensitive to different temperature indicators, while the effect estimates from certain temperature indicators did not significantly differ from those of mean temperature. Thus, more epidemiological studies should be conducted to further examine the potential susceptibility differences of different populations to various temperature indicators.

Consistent with most previous studies, our study found that cold effects on different mortality types were delayed about 1–2 days and hot effects occurred immediately. When assessing the effects of temperature on mortality, using short lags cannot completely capture both the cold effects and hot effects [17]. Cold effects may be underestimated because they usually lasted for more than a week and hot effects may be overestimated because potential mortality displacement, also referred to as harvesting, might occure in longer lags. Mortality displacement refers to the phenomenon in which a specific exposure, such as temperature, impacts already frail individuals whose deaths may have only been brought forward by a few days [33]. At least some or all of the observed effect of mortality from temperature may be explained by mortality displacement [33]. Mortality displacement has been obseverd in many studies when evaluating the effects of heat stress and cold spells on mortality [17,34]. However, the presence of mortality displacement was not consistently found in some other studies [12,33]. Moreover, a global analysis revealed that absence of displacement phenomenon of cold effects was consistently observed but varied greatly in displacement of hot effects in 12 countries/regions [9]. In our present study, both cold effect and hot effect lasted for several days and no obvious mortality displacement was observed in different mortality types, which suggests that both cold and high temperature exposure have broader impacts on the public health in Wuhan.

The results of low temperature and mortality in this study are generally comparable with previous studies in China (Table 6). For non-accidental mortality, for instance, a 1 °C decrease in mean temperature below the cold threshold in our study was associated with a 2.39% (95% CI: 1.71, 3.08) increase with a lag of 21 days, and 2.99% (95% CI: 0.85, 5.17) with a lag of 18 days in Tianjin [17], 4.3% (95% CI: 1.3, 7.5), and 4.4% (95% CI: 3.4, 5.5) in Changsha and Kunming, respectively, with a lag of 20 days [15]. Some differences, however, were that the hot effects on different mortality types were much stronger in Wuhan, which can be explained by the very high hot thresholds of mean temperature (above 30 °C, which were approximately equal to the 90th percentile of temperature distribution during the study period) due to acclimatization to local extremely hot summers, strong urban heat island effects, and high housing density in inner central urban areas. Even so, it was notable that the range from cold thresholds to minimum temperature in our study were much wider than that from hot thresholds to maximum temperature, and in this sense, extremely cold temperatures may make comparable contributions to mortality risk with extremely hot temperatures.

Compared to a substudy of PHEWE project, in which Baccini et al. [35] assessed the impact of summer heat on mortality in 15 European cities over the 1990s, our study showed a much steeper slope above threshold, which indicated higher mortality risk associated with summer heat. As previous studies noted, heat-related mortality risks varied greatly from city to city [9,36], and people in developing countries are more susceptible to heat-related mortality than developed countries due to limited adaptive capacity [2]. Also, previous evidence suggested that the elderly appeared to be at higher risk than younger populations during hot days [37], and the elderly aged over 65 accounted for 12.7% of the total population in this study. An ageing population, very hot summers, and high housing density will increase vulnerability to summer weather [38], which may contribute a lot to strong hot effects in our study area. Previous epidemiologic evidence indicated that air pollution, such as PM_10_ and O_3_, may modify the association between temperature and mortality, while the modified effects were not consistent in different studies [39]. Basu reviewed epidemiological studies from 2001 to 2008 and found that this effect modification by air pollutants was relatively small, and there was clearly an independent effect of both temperature and air pollution on mortality [39]. Additionally, a number of recent studies demonstrated that temperature-related mortality risks remained almost unchanged with and without adjusting for air pollutants [9], as well as mortality effects of heat waves [40], and cold spells [41]. Moreover, the relationship between mortality and temperature was not likely to have been substantially confounded by the effects of air pollution since the effects of air pollution on mortality were much lower than the effects of temperature [41]. Thus, we did not consider the potential interactive effects between air pollution and temperature in the present study.

Some limitations should be mentioned. First, the daily mortality data was obtained from only one district of Wuhan City, which may not completely capture the mortality effects of temperature in the whole city. Nevertheless, extreme temperature-related mortality may vary greatly within a large city [42]. Thus in our study, relatively concentrated living area, similar living conditions, and a relatively long study period may enhance the assessment precision of our study and the applicability of our results to local decision-making guidance. Second, the relatively small number of deaths due to cause-specific diseases may on one hand have limited our ability to detect tenuous differences in associations between temperature and cause-specific mortality but would not substantially affect our main findings. On the other hand, we did not use methods that could deal directly with excess zeros for respiratory and IHD mortality, such as zero-inflated Poisson models. This may thus result in some potential distortion when assessing temperature-mortality relationships. In addition, environmental exposure factors (temperature, relative humidity, and ambient pollutants) in the present study were monitored in fixed monitoring sites and cannot completely represent the actual exposure of individual level; therefore, there might exist some inevitable assessment error.

## 5. Conclusions

This study examined the effects of temperature on cause-specific mortality in Wuhan, China. The relationships between temperature and cause-specific mortality were non-linear and U-shaped. Both cold and hot temperatures were associated with increased risk in cause-specific mortality. Cold effects were delayed, while hot effects appeared immediately, and both cold and hot effects lasted for several days. These findings suggest that prevention of cold- and hot-related mortality has great public health potential and may have implications for developing intervention strategies to reduce temperature-related mortality for local government.

## Figures and Tables

**Table 1:** Descriptive statistics of daily cause-specific mortality, weather conditions, and air pollutants in Jiang’an District of Wuhan, China, 2003–2010.

Variable	Mean ± SD	Minimum	P_25_	P_50_	P_75_	Maximum
Daily death						
Non-accidental	11.2 ± 4.0	1	8	11	14	34
Cardiovascular	5.2 ± 2.7	0	3	5	7	23
Stroke	3.1 ± 2.0	0	2	3	4	17
IHD	1.3 ± 1.3	0	0	1	2	7
Respiratory	1.1 ± 1.1	0	0	1	2	7
Weather conditions						
Relative humidity (%)	71.3 ± 12.6	21	63	72	80	97
Temperature(°C)						
Maximum	22.2 ± 9.7	−1.9	14.3	23.7	30.5	39.6
Mean	17.9 ± 9.4	−2.7	9.5	19.2	25.9	35.8
Minimum	14.6 ± 9.3	−5.2	6.5	15.7	22.7	32.3
Air pollutants (μg/m^3^)						
PM_10_	115.0 ± 60.0	10.5	70.0	105.0	148.0	600.0
SO_2_	50.2 ± 33.7	1.0	26.0	42.0	66.0	260.5
NO_2_	57.6 ± 25.3	12.0	38.4	52.8	72.8	288.0

**Table 2:** Spearman’s correlation coefficients of weather conditions and air pollutants in Jiang’an District of Wuhan, China, 2003–2010 *.

	Mean Temperature	Minimum Temperature	Relative Humidity	PM_10_	SO_2_	NO_2_
Maximum temperature	0.983	0.947	−0.229	−0.169	−0.227	−0.196
Mean temperature		0.987	−0.163	−0.226	−0.284	−0.258
Minimum temperature			−0.079	−0.274	−0.334	−0.315
Relative humidity				−0.241	−0.286	−0.194
PM_10_					0.632	0.721
SO_2_						0.693

* *p* < 0.001 for all correlation coefficients.

**Table 3:** Akaike information criteria (QAIC) values of non-threshold and double-threshold DLNM models predicted by different temperature metrics.

DLNM Type	Temperature Metrics	QAIC Value				
		Non-Accidental	Cardiovascular	Respiratory	Stroke	IHD
Non-threshold ^a^	Maximum temperature	15,354.85	13,057.47	7587.25	11,367.90	8433.61
	Mean temperature	15,323.12	13,043.74	7584.90	11,359.48	8432.13
	Minimum temperature	15,327.35	13,048.02	7590.40	11,370.68	8434.02
Double-threshold ^b^	Maximum temperature	15,336.71	13,038.42	7566.27	11,342.09	8410.93
	Mean temperature	15,315.91	13,034.61	7568.58	11,339.88	8407.63
	Minimum temperature	15,315.93	13,040.98	7572.09	11,346.56	8408.51

Notes: ^a^ Using “natural cubic spline-natural cubic spline” DLNM with smoothing of 6 degrees of freedom for temperature and 4 degrees of freedom for lag; ^b^ Using “double-threshold-natural cubic spline” DLNM with smoothing of 4 degrees of freedom for lag.

**Table 4:** Cold and hot thresholds (°C) used by the double-threshold-natural cubic spline DLNM.

Threshold Type	Temperature Metrics	Mortality Type				
		Non-Accidental	Cardiovascular	Respiratory	Stroke	IHD
Cold threshold (°C)	Maximum temperature	22.3	20.2	19.4	24.6	3.1
	Mean temperature	18.1	15.6	14.6	20.2	3.5
	Minimum temperature	13.9	13.3	11.0	18.1	−1.5
Hot threshold (°C)	Maximum temperature	34.7	36.1	34.6	37.1	34.4
	Mean temperature	31.7	31.4	31.2	32.2	30.0
	Minimum temperature	28.8	28.9	28.0	29.2	26.6

**Table 5:** The cumulative cold and hot effects of mean temperature on cause-specific mortality along the lag days, using a double-threshold-natural cubic spline DLNM with 4 df natural cubic spline for lag.

Effect	Lag (Days)	Percent Increase in Mortality (95% CI)				
		Non-Accidental	Cardiovascular	Respiratory	Stroke	IHD
Cold effect ^a^	0	−0.22(−0.63, 0.20)	0.49(−0.16, 1.14)	−0.21(−1.59, 1.19)	0.17(−0.56, 0.91)	−1.97(−5.92, 2.16)
	0–2	0.17 (−0.45, 0.80)	1.41 (0.44, 2.39)	0.32 (−1.75, 2.43)	1.12 (0.01, 2.25)	−0.81 (−6.67, 5.41)
	0–7	1.73 (1.10, 2.37)	2.95 (1.96, 3.95)	2.08 (−0.02, 4.23)	3.20 (2.06, 4.36)	8.11 (1.59, 15.06)
	0−14	2.25 (1.54, 2.98)	3.59 (2.50, 4.71)	2.62 (0.27, 5.03)	3.44 (2.16, 4.75)	15.14 (7.75, 23.05)
	0–21	2.39 (1.71, 3.08)	3.65 (2.62, 4.69)	3.87 (1.57, 6.22)	3.13 (1.88, 4.38)	21.57 (12.59, 31.26)
Hot effect ^b^	0	7.64 (4.51, 10.86)	8.43 (4.48, 12.53)	10.94 (2.45, 20.13)	15.50 (7.83, 23.71)	4.30 (−0.27, 9.07)
	0–2	17.67 (12.63, 22.94)	21.55 (15.05, 28.41)	19.37 (5.98, 34.47)	36.94 (24.16, 51.03)	11.62 (4.32, 19.43)
	0–7	25.18 (18.74, 31.96)	34.10 (25.63, 43.16)	24.27 (7.55, 43.59)	59.1 (41.81, 78.5)	17.00 (7.91, 26.87)
	0−14	23.03 (14.68, 31.99)	28.97 (18.06, 40.89)	46.94 (23.49, 74.85)	65.88 (40.87, 95.32)	8.59 (−1.82, 20.11)
	0–21	24.74 (13.66, 36.89)	30.17 (15.78, 46.35)	56.74 (24.28, 97.69)	61.59 (27.87, 104.19)	9.83 (−2.85, 24.17)

Notes: ^a^ The percent increase in mortality for a 1 °C of temperature decrease below the cold thresholds (18.1 °C for non-accidental, 15.6 °C for cardiopulmonary, 14.6 °C for respiratory, 20.2 °C for stroke, and 3.5 °C for IHD mortality); ^b^ The percent increase in mortality for a 1 °C of temperature increase above the hot thresholds (31.7 °C for non-accidental, 31.4 °C for cardiopulmonary, 31.2 °C for respiratory, 32.2 °C for stroke, and 30.0 °C for IHD mortality).

**Table 6:** Study results of cold- and heat-related mortality in other Chinese cities using double-threshold DLNM.

Location	Date Range	Study Design	Temperature Threshold		Main Results	
			Low (°C)	High (°C)	Cold Effect ^a^	Hot Effect ^b^
Tianjin	2005–2007	case-crossover	0.8	24.9	2.99 (0.85, 5.17) ^c^	2.03 (0.70, 3.38) ^d^
Changsha	2006–2009	time-series	7	25	4.3 (1.3, 7.5) ^e^	2.0 (0.3, 3.7) ^f^
Kunming	2006–2009	time-series	15	19	4.4 (3.4, 5.5) ^e^	1.7 (0.4, 3.0) ^f^
Guangzhou	2006–2010	time-series	13	26	9.4 (7.6, 11.3) ^e^	2.9 (2.0, 3.9) ^f^
Zhuhai	2006–2010	time-series	15	26	10.3 (7.5, 13.3) ^e^	2.3 (0.4, 4.2) ^f^

Notes: ^a^ The percent increase in mortality for a 1 °C of temperature decrease below the cold thresholds; ^b^ The percent increase in mortality for a 1 °C of temperature increase above the hot thresholds; ^c^ lag 0–18; ^d^ lag 0–2; ^e^ lag 0–20; ^f^ lag 0.

## Supplementary Materials

The following are available online at www.mdpi.com/1660-4601/13/7/722/s1, Figure S1: Sensitivity analyses for total mortality using different threshold values, Figure S2: The non-linear effects of mean temperature on age-specific mortality at lag 0–21 (total mortality), Figure S3: The estimated effects of a 1 °C decrease in mean temperature below the cold threshold (above) and of a 1 °C increase in mean temperature above the hot threshold (below) on age-specific mortality over 21 days of lag, Table S1: Cold and hot thresholds of mean temperature for total mortality stratified by age.
