# Supplementary material for: The Short-Term Effect of Ambient Temperature on Mortality in Wuhan, China: A Time-Series Study Using a Distributed Lag Non-Linear Model
Source: Int J Environ Res Public Health. 2016 Jul 18;13(7):722. doi: 10.3390/ijerph13070722 (PMC4962263; doi:10.3390/ijerph13070722)
Supplement: Supplementary file 1 [file ijerph-13-00722-s001.pdf]

Supplementary Materials: The Short-Term Effect of Ambient Temperature on Mortality in Wuhan, China: A Time-Series Study Using a Distributed Lag Non-Linear Model

Yunquan Zhang, Cunlu Li, Renjie Feng, Yaohui Zhu, Kai Wu, Xiaodong Tan and Lu Ma

Table S1. Cold and hot thresholds of mean temperature for total mortality stratified by age.

Age Group	Cold Threshold (°C)	Hot Threshold (°C)
Age < 65	15.9	27.3
Age ≥ 65	18.2	32.1

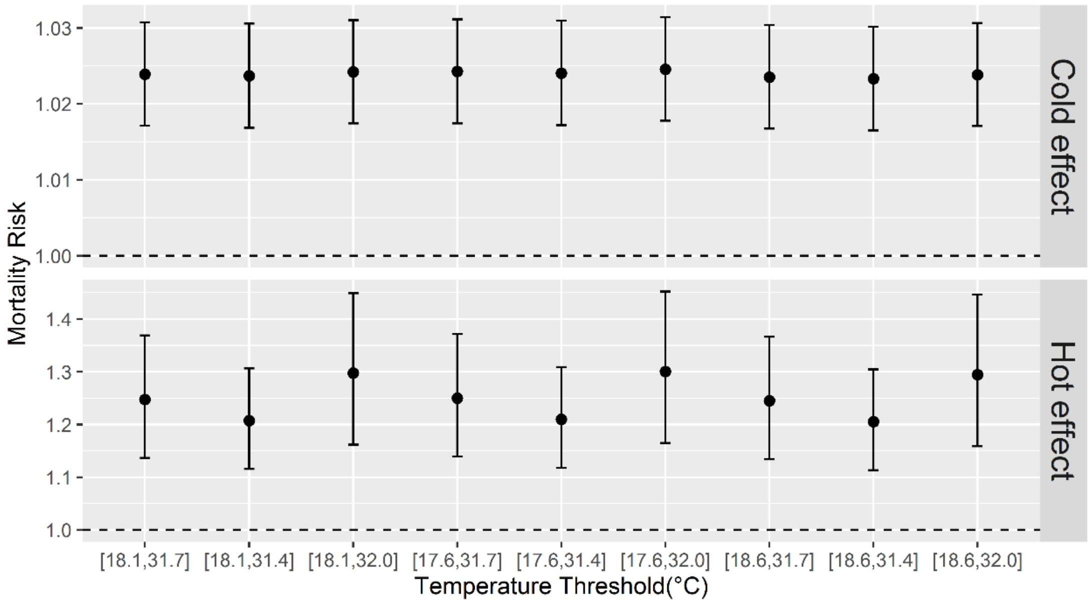

Figure S1. Sensitivity analyses for total mortality using different threshold values.

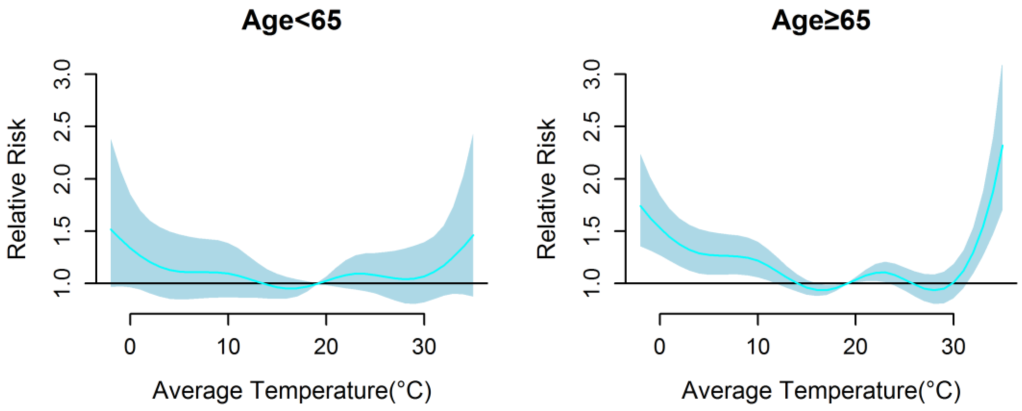

Figure S2. The non-linear effects of mean temperature on age-specific mortality at lag 0–21 (total mortality).

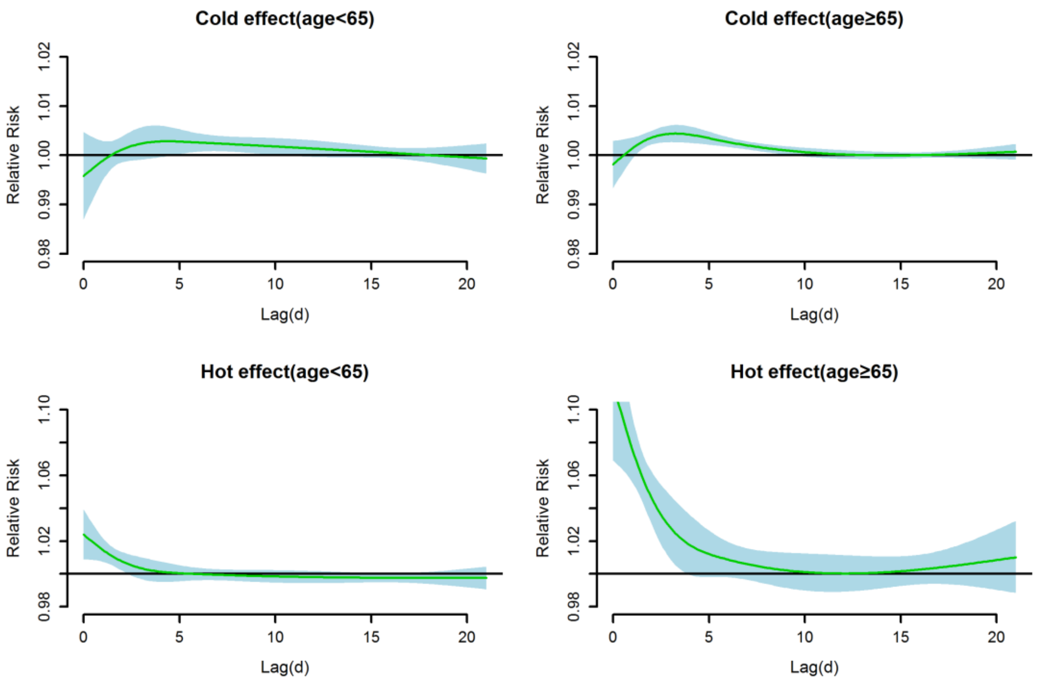

Figure S3. The estimated effects of a 1 °C decrease in mean temperature below the cold threshold (**above**) and of a 1 °C increase in mean temperature above the hot threshold (**below**) on age-specific mortality over 21 days of lag.

© 2016 by the authors. Submitted for possible open access publication under the terms and conditions of the Creative Commons Attribution (CC-BY) license (http://creativecommons.org/licenses/by/4.0/).
